# Integrated Multi-Omics Analyses Reveal That Autophagy-Mediated Cellular Metabolism Is Required for the Initiation of Pollen Germination

**DOI:** 10.3390/ijms241915014

**Published:** 2023-10-09

**Authors:** Xuemei Zhou, Qiuyu Zhang, Yuliang Zhao, Shanshan Ding, Guang-Hui Yu

**Affiliations:** College of Life Sciences, South-Central Minzu University, Wuhan 430074, China; xmzhou1988@163.com (X.Z.);

**Keywords:** autophagy, multi-omics analyses, metabolome, transcriptome, pollen germination, tobacco

## Abstract

Autophagy is an evolutionarily conserved mechanism for degrading and recycling various cellular components, functioning in both normal development and stress conditions. This process is tightly regulated by a set of autophagy-related (ATG) proteins, including ATG2 in the ATG9 cycling system and ATG5 in the ATG12 conjugation system. Our recent research demonstrated that autophagy-mediated compartmental cytoplasmic deletion is essential for pollen germination. However, the precise mechanisms through which autophagy regulates pollen germination, ensuring its fertility, remain largely unknown. Here, we applied multi-omics analyses, including transcriptomic and metabolomic approaches, to investigate the downstream pathways of autophagy in the process of pollen germination. Although *ATG2* and *ATG5* play similar roles in regulating pollen germination, high-throughput transcriptomic analysis reveals that silencing *ATG5* has a greater impact on the transcriptome than silencing *ATG2*. Cross-comparisons of transcriptome and proteome analysis reveal that gene expression at the mRNA level and protein level is differentially affected by autophagy. Furthermore, high-throughput metabolomics analysis demonstrates that pathways related to amino acid metabolism and aminoacyl-tRNA biosynthesis were affected by both *ATG2* and *ATG5* silencing. Collectively, our multi-omics analyses reveal the central role of autophagy in cellular metabolism, which is critical for initiating pollen germination and ensuring pollen fertility.

## 1. Introduction

Autophagy is an evolutionarily conserved mechanism that degrades cellular components to recycle nutrients or eliminates unwanted materials in eukaryotes. The major and best-studied type of autophagy is macroautophagy, referred to hereafter simply as autophagy. During this process, cellular materials are engulfed and sequestered within double membrane-bound autophagosomes, which are subsequently delivered to the lysosome or vacuole for degradation by proteases and hydrolases. Similar to yeast and mammals, plant autophagy is tightly controlled by numerous autophagy-related (ATG) proteins. More than 30 ATGs have been identified in different plants [1,2,3], and they can be classified into five functional complexes including the regulation complex, ATG9 complex, the Ubiquitin-like ATG12 and ATG5 conjugation pathway, the Phosphoinositide-3-kinase (PI3K) complex, and the Ubiquitin-like ATG8 and PE conjugation pathway [4]. Recent researches demonstrated that various organelles, including peroxisomes [5], chloroplasts [6], and various macromolecular components such as starch [7] and lipids [8], can be degraded through autophagy, which is critical for plant growth and development under both stress and normal conditions.

However, the molecular and metabolic pathways of autophagy in these physiological processes are still largely unknown. Omics studies have been conducted to investigate the role of autophagy in cellular metabolism *atg* mutants grown under normal and nutrient starvation conditions [8,9,10], indicating the central role of autophagy in catabolism and lipid metabolism. Metabolomics studies revealed significant alterations in the levels of free amino acids and secondary metabolites. Altered lipid levels were also found in the *atg* mutants through lipidomic analyses [8]. The role of autophagy in lipid metabolism was further supported by proteomic analysis, which revealed elevated protein levels of peroxisomal and endoplasmic reticulum (ER) proteins in *atg5* mutants. These proteins were associated with long-chain fatty acid synthesis and β-oxidation [9]. Multi-omics studies on maize *atg12* mutants, grown under nitrogen-rich and -deficient conditions, further confirmed the impact of autophagy on cellular metabolism. The levels of metabolites related to lipid/fatty acid metabolism and secondary metabolites associated with flavonoids, anthocyanins, and antioxidants were significantly altered in *atg12* mutant. Additionally, several cellular organelles, including peroxisomes, Golgi bodies, ER, ribosomes, and protein degradation complexes such as proteasomes, were targeted for autophagic clearance [10].

Our recent study revealed that autophagy-mediated compartmental cytoplasm clearance is critical for pollen germination [11]. We observed a dramatic increase in autophagic activity during the initial stage of pollen germination. Inhibition of autophagy, achieved by silencing autophagy-specific genes—*ATG2* and *ATG5*, which belong to different ATG function complexes and serve distinct roles in autophagosome formation—resulted in similar defects in pollen germination and cytoplasmic clearance in the germination aperture. This discovery unveils a novel mechanism of compartmentalized autophagy control in male fertility, specifically through cytoplasmic clearance. Lipidomic analyses revealed alterations in the profiles of stored and signaling lipids in autophagy-deficient pollen [11]. However, the downstream pathways of autophagy in regulating pollen germination are still waiting to be elucidated. To gain insight into the comprehensive effects of autophagy on the regulation of pollen germination in tobacco, we conducted a comprehensive metabolomic, proteomic, and transcriptomic analysis of *ATG2* and *ATG5*- silenced pollen, which allowed us to reveal connections between autophagy and downstream pathways. Combing the results from the metabolomics, lipidomics, transcriptomics, and proteomics, we discovered that pathways related to amino acid and lipid catabolic processes during the initiation stages of pollen were strongly influenced by autophagy, which is critical for promoting pollen germination and ensuring pollen fertility.

## 2. Results

### 2.1. Global Impact of Silencing of ATG2 and ATG5 on Pollen Transcriptome

**Figure 1:**
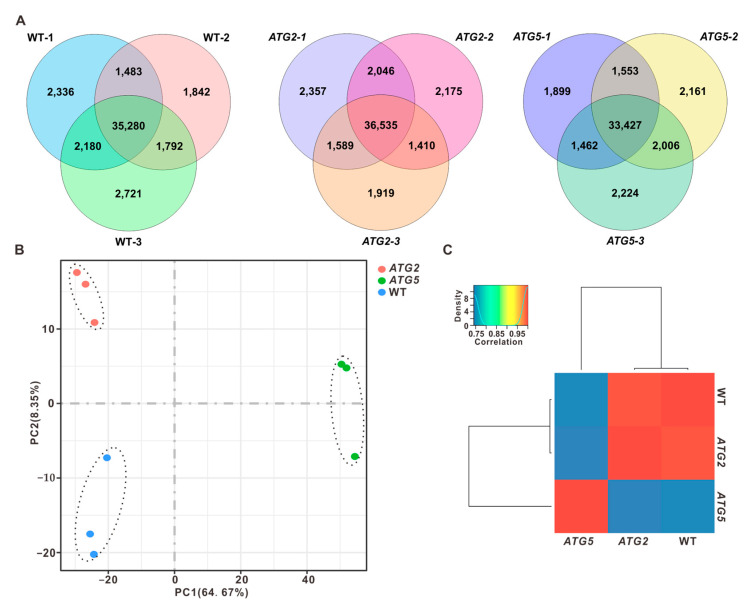
Global transcriptome analysis of WT, *ATG2*-silenced, and *ATG5*-silenced pollen (**A**). Overlap analysis of detected genes in three independent biological replicates. (**B**). Principal component analysis of the transcriptomes of WT, *ATG2*-silenced, and *ATG5*-silenced pollen. (**C**). Clustering analysis the transcriptome of WT, *ATG2*-silenced, and *ATG5*-silenced pollen according to the Pearson’s correlation efficient.

Our recent studies demonstrated that silencing of *ATG2* or *ATG5* leads to the defects in pollen germination [11]. In order to investigate the impact of autophagy on pollen germination, we first investigated how silencing of *ATG2* and *ATG5* affects the pollen transcriptome. Pollen from wild type (WT), *ATG2*-silenced, and *ATG5*-silenced plants were collected for RNA sequencing (RNA-seq). Three independent biological replicates were performed for each genotype. Thus, we constructed nine RNA-seq libraries and generated over 20 million read pairs for each library. Following the quality assessment of the RNA-seq data, the clean reads of each sample were then mapped onto the tobacco genome and reference transcripts (Ntab-TN90 reference Annotation Release 100). This resulted in the detection of 38,000–43,000 genes (fragments per kilobase of transcript per million mapped reads [FPKM] > 0) in different samples (Figure 1A and Table S1). Overall, the detected genes from the same genotype but different biological repeats were highly correlated (r ≥ 0.98; Figure S1), indicating that our transcriptome data are of high quality and reliable for subsequent analysis.

To compare the transcriptome of WT, *ATG2*-silenced, and *ATG5*-silenced pollen, RNA-seq data were subjected to principal component analysis (PCA). The results revealed that PC1 efficiently distinguished the *ATG5* group from the WT and *ATG2* groups, but did not separate *ATG2* from WT. On the other hand, both PC1 and PC2 were able to distinguish the *ATG2* group from WT (Figure 1B). To further access the impact of silencing of *ATG2* and *ATG5* on the pollen transcriptome, pairwise correlations were analyzed. Consistent with the PCA results, the transcriptome of *ATG2*-silenced pollen clustered with WT, but was distinct from the *ATG5* group, suggesting that silencing *ATG5* has a stronger influence on the pollen transcriptome compared to silencing *ATG2* (Figure 1C).

### 2.2. Different Impact of Silencing of ATG2 and ATG5 on Pollen Transcriptome

**Figure 2:**
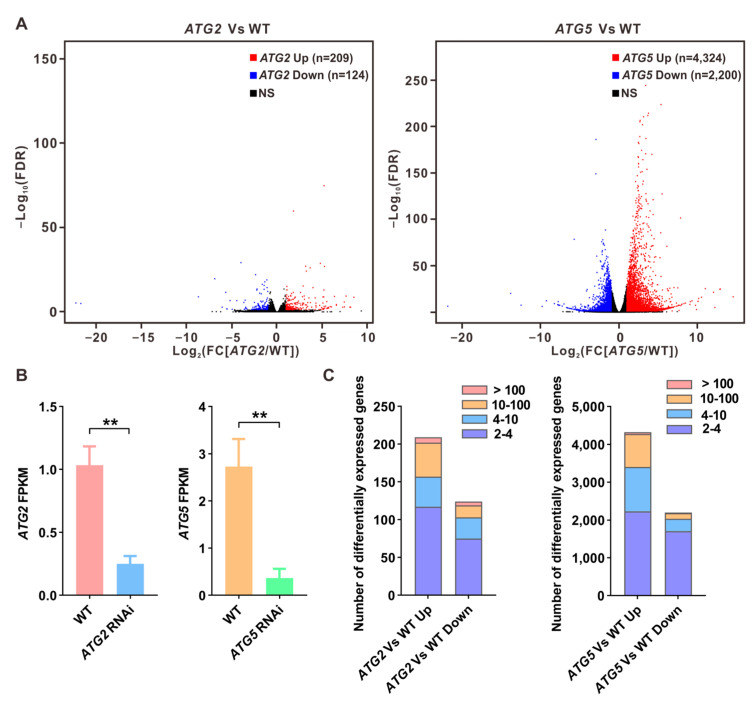
Identification of differentially expressed genes between WT and *ATG*-silenced pollen. (**A**). Volcano plots displaying differentially expressed genes between *ATG2*-silenced and WT pollen or *ATG5*-silenced and WT pollen. Red dots indicate upregulated genes, and blue dots indicate downregulated genes. (**B**). FPKM values of *ATG2* and *ATG5* in WT and their respective RNAi lines. (**C**). Number of differentially expressed genes between *ATG2*-silenced and WT pollen or *ATG5*-silenced and WT pollen. Fold changes are indicated with the distinct colors. Student’s t test, ** *p* < 0.05.

Both PCA and pairwise correlation analyses revealed that silencing of *ATG2* and *ATG5* has a distinct impact on the pollen transcriptome (Figure 1B; C). To further compare the differential influences of silencing *ATG2* and *ATG5* on the pollen transcriptome, we identified differently expressed genes between *ATG2*-silenced and WT pollen, as well as between *ATG5*-silenced and WT pollen, respectively (Figure 2A). To verify the high reliability of the RNA-seq data, we first analyzed the expression levels of *ATG2* and *ATG5* based on the RNA-seq data. The results confirmed that the expression levels of *ATG2* and *ATG5* are significantly downregulated in their respective silencing lines (Figure 2B). Next, we found that in *ATG2*-silenced pollen compared to WT, 209 genes were significantly upregulated (fold change [FC][*ATG2*/WT] > 2, false discovery rate [FDR] < 0.05), while 124 genes were significantly downregulated (FC[*ATG2*/WT] < 0.5, FDR < 0.05) (Figure 2A,C and Supplementary Data S1). Consistent with the PCA results, the number of differently expressed genes between *ATG5*-silenced and WT pollen was significantly larger than that between *ATG2*-silenced and WT pollen. Specifically, we observed that 4324 genes were significantly upregulated (FC[*ATG5*/WT] > 2, FDR < 0.05), and 2200 genes were significantly downregulated in *ATG5*-silenced pollen compared with the WT (FC[*ATG5*/WT] < 0.5, FDR < 0.05) (Figure 2A,C and Supplementary Data S1). All these data collectively indicate that silencing of *ATG5* has a greater impact on the transcriptome than compared to *ATG2*.

### 2.3. The Levels of Transcripts Linked to Metabolic Process Changed in ATG2-Silenced and ATG5-Silenced Pollen

**Figure 3:**
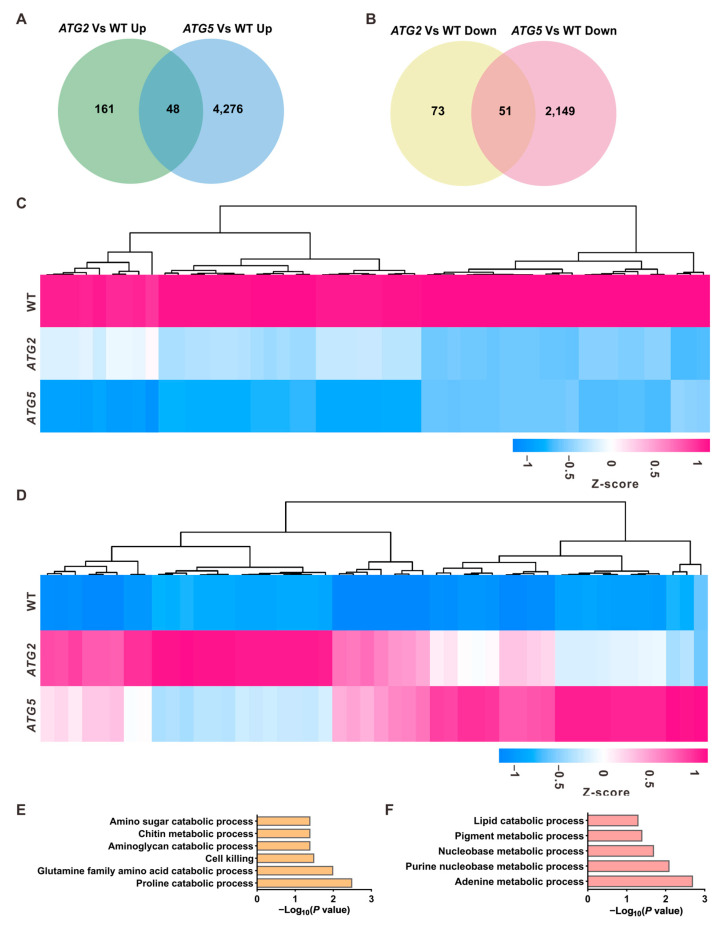
Identification of downstream pathways of autophagy in the process of pollen germination (**A**,**B**). Overlap analysis of upregulated genes (**A**) and downregulated genes (**B**) between *ATG2*/WT and *ATG5*/WT. (**C**,**D**). Heat maps showing the expression levels of the commonly downregulated (**C**) and commonly upregulated genes (**D**) in *ATG2*-silenced and *ATG5*-silenced pollen. (**E**,**F**). GO enrichment analysis of the commonly downregulated (**E**) and upregulated genes (**F**) in *ATG2*-silenced and *ATG5*-silenced pollen.

Despite the distinct impacts of silencing *ATG2* and *ATG5* on the pollen transcriptome, they exhibited similar effects on pollen germination. Both silencing of *ATG2* and *ATG5* blocked the pollen germination [11], providing an opportunity to investigate downstream pathways of autophagy responsible for the initiation of pollen germination at the transcriptional level. Consistent with the similar defects in germination of *ATG2*-silenced and *ATG5*-silenced pollen grains, a substantial number of differentially regulated genes were overlapped between *ATG2*/WT and *ATG5*/WT (Figure 3A,B). We then turned our focus to the detailed roles of these commonly differentially regulated genes in the process of pollen germination (Figure 3C,D). Gene Ontology (GO) analysis was performed for the commonly downregulated and upregulated genes in *ATG*-silenced pollen, respectively. GO biological process enrichment analysis revealed distinct molecular pathways enriched in downregulated and upregulated genes (Figure 3E,F). In *ATG*-silenced pollen, downregulated genes were mainly enriched in metabolic processes such as the proline catabolic process (*p* = 3.3 × 10^−^^3^) and glutamine family amino acid catabolic process (*p* = 2.1 × 10^−^^3^). On the other hand, upregulated genes showed enrichment in the adenine metabolic process (*p* = 2.1 × 10^−^^3^) and lipid catabolic process (*p* = 4.9 × 10^−^^2^). These findings suggest that amino acid and lipid catabolic processes are potential downstream pathways regulated by autophagy during pollen germination.

### 2.4. Autophagy Has Differential Effects on Gene Expression at Both the mRNA and Protein Levels

**Figure 4:**
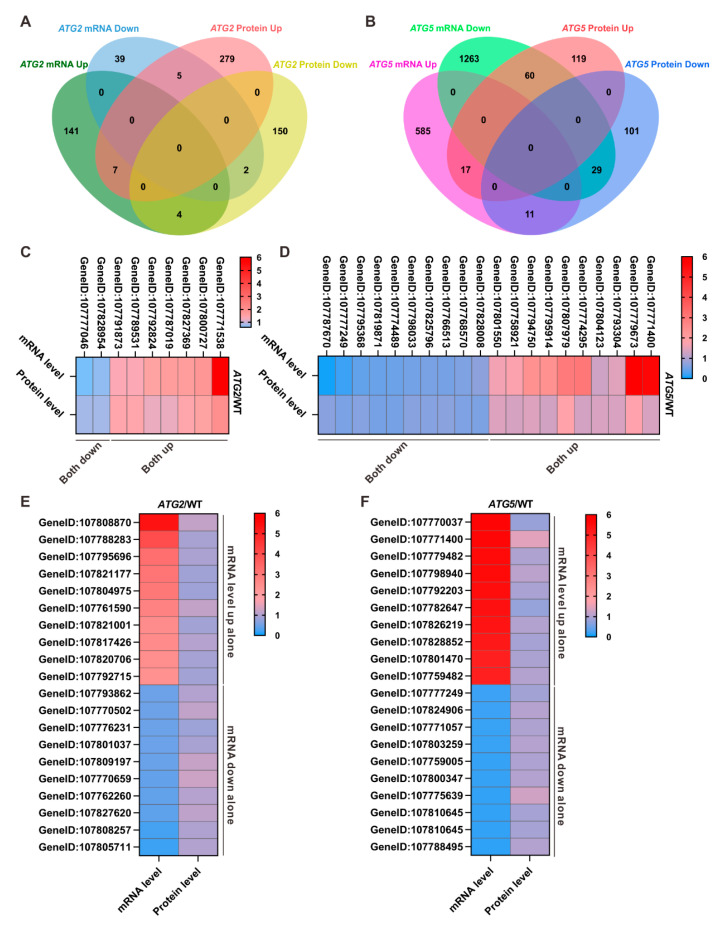
Comparisons between differentially regulated transcripts and proteins in *ATG2*-silenced and *ATG5*-silenced pollen (**A**,**B**). Overlap analysis of differentially regulated transcripts and proteins in *ATG2*-silenced (**A**) and *ATG5*-silenced (**B**) pollen. (**C**,**D**). Heat maps showing differentially expressed genes that consistently change at mRNA and protein levels in *ATG2*-silenced (**C**) and *ATG5*-silenced (**D**) pollen. (**E**,**F**). Heat maps showing genes that are differentially regulated at both mRNA and protein levels in *ATG2*-silenced (**E**) and *ATG5*-silenced (**F**) pollen.

Our recent proteomic analysis has revealed a profound impact on the proteome due to the silencing of *ATG* genes [11]. In order to establish correlations between changes at the mRNA and protein levels, we initially generated a dataset comprising genes detected in both proteome and transcriptome data (Supplementary Data S2). Utilizing this dataset, we conducted comparisons between differentially expressed proteins and genes in *ATG*-silenced pollen. Among the 7834 genes, we observed that 627 genes exhibited expression differences at either the mRNA or protein level in *ATG2*-silenced pollen, while 2185 genes displayed changes at either the mRNA or protein level in *ATG5*-silenced pollen (Figure 4). Surprisingly, only a small subset of genes exhibited similar changes at the protein and mRNA level. Specially, only 9 genes in *ATG2*-silenced pollen and 46 genes in *ATG5*-silenced pollen consistently displayed altered expression trends at both the protein and mRNA levels. In contrast, the majority of genes exhibited differential alteration trends at the protein and mRNA levels in *ATG*-silenced pollen. In *ATG2*-silenced pollen, we identified 180 genes that displayed changes exclusively at the mRNA level, with no corresponding changes at the protein level, and 429 genes that showed changes only at the protein level, without corresponding mRNA-level changes. Similarly, in *ATG5*-silenced pollen, 1848 genes displayed changes exclusively at the mRNA level, while 220 genes showed changes solely at the protein level (Figure 4). Taken together, all these data collectively suggest that genes downstream of autophagy undergo differential regulation at the mRNA and protein level.

### 2.5. Downregulation of ATGs Leads to the Decrease in Metabolic Level in Pollen

**Figure 5:**
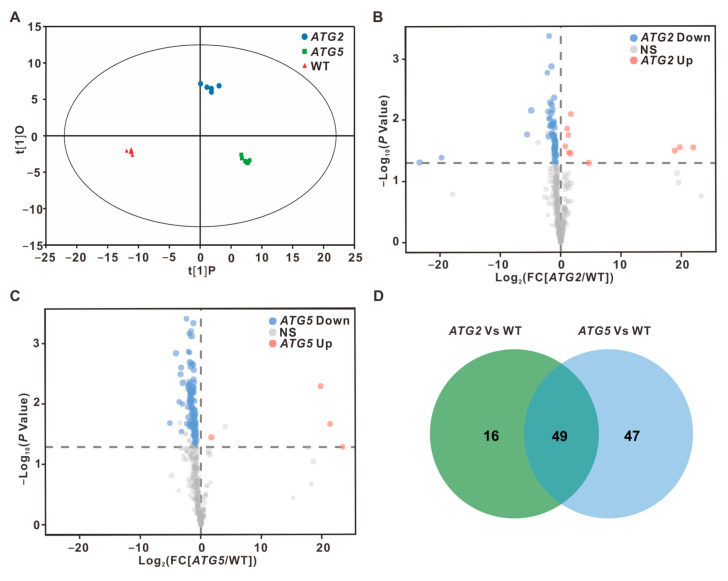
Silencing *ATGs* results in the decreased metabolite levels in pollen (**A**). OPLS-DA of metabolite levels. The ellipse represents the Hotelling T2 with 95% confidence. (**B**,**C**). Volcano plots showing metabolites with different abundances between WT and *ATG*-silenced pollen. Blue dots indicate downregulated metabolites in *ATG*-silenced pollen, while pink dots indicate upregulated metabolites in *ATG*-silenced pollen. (*t*-test, *p* < 0.05). (**D**). Venn diagrams showing the overlaps of metabolites with significantly different contents in *ATG*-silenced pollen.

Autophagy has been linked to nutrient recycling under starvation conditions [12,13,14]. However, the influence of autophagy on central metabolism under normal developmental conditions are still largely unknown. A comparative transcriptome analysis revealed the significant changes in the expression levels of numerous genes related to various metabolic processes in *ATG*-silenced pollen. To further investigate how autophagy affects pollen metabolism, we performed a comprehensive metabolite profiling study to examine the metabolic contents of *ATG*-silenced pollen in comparison to the WT pollen using GC-TOF MS, which allowed for the quantitative analysis of primary metabolites. The contents of 374 metabolites in WT, *ATG2*-silenced, and *ATG5*-silenced pollen were quantified based on the combined analysis results from six to eight independent biological replicates. Orthogonal projections to latent structures-discriminant analysis (OPLS-DA) displayed a clear separation between the WT and *ATG* RNAi lines, suggesting that silencing of either *ATG* gene leads to changes in the concentrations of metabolites when compared to the WT (Figure 5A).

To further investigate the impact of silencing of *ATG* genes on the pollen metabolome, the metabolites that exhibited different levels between *ATG2* and WT or between *ATG5* and WT were screened. Our analysis revealed that in *ATG2*-silenced pollen compared to WT, the contents of 10 metabolites increased significantly, while the contents of 55 metabolites decreased significantly. Consistent with the findings from the transcriptome analysis, we observed more pronounced metabolic changes in *ATG5*-silenced pollen. Specifically, there was a significant increase in 4 metabolites and a significant decrease in 92 metabolites compared to the WT (Figure 5B,C and Supplementary Data S3).

### 2.6. Metabolic Pathways Impacted by Autophagy

**Figure 6:**
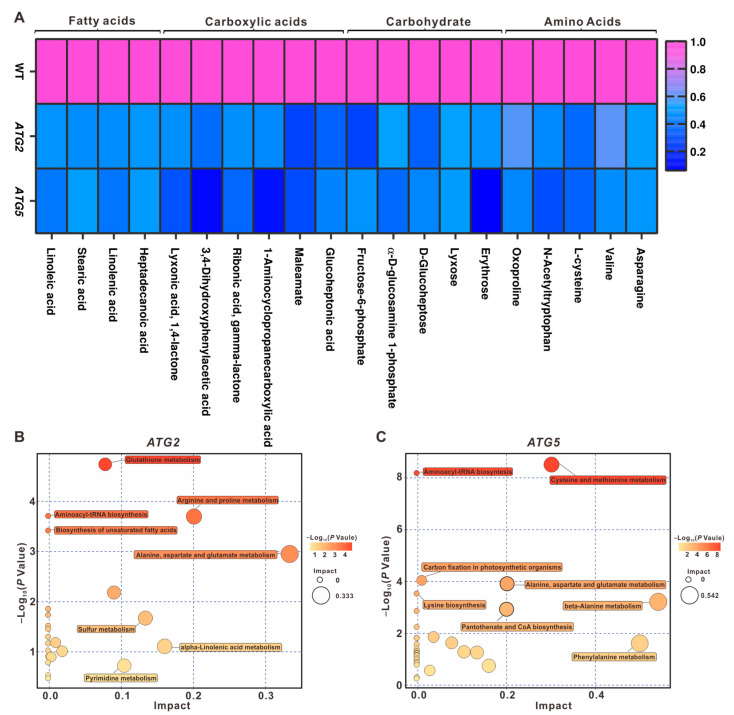
Significant impact of *ATG* downregulation on downstream metabolic pathways (**A**). Heat map depicting the relative metabolite contents. (**B**,**C**). Metabolic pathways regulated by *ATG2* (**B**) or *ATG5* (**C**).

To elucidate the metabolic pathways regulated by *ATG2* and *ATG5*, the common metabolites regulated by both *ATG2* and *ATG5* were identified. A statistically significant overlap was observed among the metabolites that were downregulated in *ATG2*-silenced and *ATG5*-silenced pollen (Figure 5D). Notably, we observed a significant decrease in the levels of free amino acids in *ATG2*-silenced and *ATG5*-silenced pollen compared with the WT. Moreover, when comparing *ATG5*-silenced pollen to *ATG2*-silenced pollen, we noted a more substantial reduction in the levels of free amino acids (Figure 6A). Notably, the level of valine exhibited a significant decrease (Figure 6A), which has been demonstrated to function as an electron donor for the tricarboxylic acid (TCA) cycle and mitochondria electron transport chain under carbon starvation [15]. In addition, reduced steady-state levels of other organic compounds such as carbohydrates, fatty acids, and carboxylic acids were also observed in *ATG*-silenced pollen (Figure 6B,C). These results indicate that downregulation of either *ATG* gene results in a reduced central metabolic level in pollen, suggesting the conserved role of autophagy in central metabolism under normal developmental conditions as that in stress conditions [8].

When the metabolites significantly affected in *ATG2*-silenced and *ATG5*-silenced pollen were grouped based on their metabolic pathways, we discovered dramatic impacts on metabolic pathways in autophagy-deficient pollen. While the majority of metabolic pathways exhibited differential enrichment in *ATG2*-silenced and *ATG5*-silenced pollen (Figure 6B,C), two pathways were consistently impacted in both cases: alanine, aspartate, and glutamate metabolism, as well as aminoacyl-tRNA biosynthesis. This observation suggests direct connections between autophagy and these two pathways. 

## 3. Discussion

### 3.1. Differential Regulation of Downstream Genes of Autophagy at mRNA and Protein Levels

Comparative proteomic and transcriptomic analyses reveal a notable characteristic: numerous genes display differential abundances at both the mRNA and protein levels in *ATG*-silenced pollen. In *ATG2*-silenced pollen, hundreds of differentially expressed genes were identified, yet only 9 of them displayed the similar changing trend at the protein level. Moreover, a limited number of differentially expressed genes were commonly regulated at both the mRNA and protein levels. All these data suggest that defects in autophagy have distinct impacts on the cellular mRNA and cellular protein levels. This observation is consistent with the findings from Arabidopsis *atg5* mutants, where the changes in the protein levels of most genes did not correlate with alterations in the mRNA levels [9]. It is proposed that genes may be regulated at the mRNA level (involving mRNA synthesis and degradation), protein level (involving protein synthesis and degradation), and within the autophagy process itself or through a combination of these processes. There is growing evidence supporting the crucial roles of autophagy in both RNA decay and protein degradation [16,17]. Our present comparative proteomic and transcriptomic analyses suggest that relying solely on RNA or protein level analysis may not be sufficient for accurately studying downstream autophagy pathways or establishing precise connections between autophagy and specific cellular events. Integrated multi-omics approaches are more effective for comprehending the role of autophagy in plants and exploring downstream pathways in future studies.

### 3.2. Autophagy-Dependent Cellular Metabolism Is Critical for Promoting Pollen Germination

Both transcriptomic and metabolomic analyses of pollen grains and pollen tube at different developmental stages support the idea that mature pollen already contains most transcripts involved in different metabolic pathways for pollen germination and that dramatic metabolic changes occur during the process to facilitate pollen tube emergence [18,19,20,21]. These studies show that most metabolites related to the carbohydrate and energy pathways, such as carbohydrate metabolism, TCA cycle, pyruvate utilization, and glycolysis, are present at relatively high levels in mature pollen grains compared to other tissues. Furthermore, metabolic pathways related to starch and fatty acid degradation are also activated in the initial stage of pollen germination. These findings indicate that pollen germination and tube growth are high energy-consumption processes and that high metabolic activity is required for the initiation of pollen germination [22,23,24,25,26]. While mature pollen stored carbohydrates and other storage components can support pollen germination, rapid pollen tube growth necessitates uptake carbohydrates and other stored components from surrounding tissues as an energy source [26,27]. Metabolite profiling analysis of male gametophytes at different developmental stages also supports this viewpoint [19]. The levels of storage components, such as sucrose and triradylglycerol, were observed to peak in mature pollen during the male gametophyte development and decrease as pollen germination occurs. This suggests that metabolites derived from these storage molecules could be recycled to support pollen germination [19].

Our integrated multi-omics analysis results reveal that autophagy, in collaboration with several metabolic pathways, plays a critical role in recycling the storage components of mature pollen during the initial stage of pollen germination (Figure 6 and Figure S3). When comparing the transcriptomes and metabolomes of WT and autophagy-suppressed pollen, we observed a significant downregulation of transcripts and metabolites in metabolic pathways, such as amino acid metabolism, carbohydrate metabolism, and fatty acid metabolism. This suggests that autophagy-dependent metabolic regulation is critical for pollen germination. Research focusing on sucrose transport proteins, invertases, and monosaccharide transporters also supports the active uptake of carbohydrates as an energy source for pollen tube growth [28,29]. In tobacco, extracellular invertase and monosaccharide transporters, NtMST2, and NtMST3 were found to be crucial for pollen germination and tube growth, potentially through the supply of carbohydrates. Inhibiting invertase activities using a chemical invertase inhibitor or the expressing of an invertase inhibitor NtCIF resulted in a reduced sucrose uptake and compromised pollen germination [22]. The role of sucrose uptake in pollen germination has also been observed in Arabidopsis. One example is AtSUC1, which encodes a sucrose transporter and is localized to the plasma membrane in pollen tubes. While mutation in AtSUC1 did not affect pollen viability, pollen germination was significantly decreased in *suc1* mutants when compared with the WT plants [29]. In addition, proline has been found to be essential for pollen development and germination through the analysis of Δ1-pyrroline-5-carboxylate synthetase (P5CS) and Δ1-pyrroline-5-carboxylate reductase (P5CR), critical enzymes in proline synthesis [30]. Although the role of specific metabolites such as carbohydrates and fatty acids in pollen germination has not been directly tested, dynamic changes in fatty acid composition during pollen germination have been revealed in various plants [31]. Our findings provide evidence that during pollen germination, autophagy not only mediates selective cytoplasmic clearance but also regulates the activity of these well-known critical metabolic processes. It is worth testing the exact role of these metabolic pathways in pollen germination and identifying the direct connection between the metabolites and pollen germination in future studies.

### 3.3. Differences and Similarities between Tobacco and Arabidopsis atg5 Transcriptomes

At present, no transcriptome data of autophagy-deficient pollen are available in Arabidopsis and other model plants, which do not allow us to compare the differences in the transcriptome of autophagy-deficient pollen from different plants. However, transcriptome analyses have been performed in Arabidopsis *atg5* rosettes under stress conditions [32], which gives us an opportunity to compare the similarities and differences in the impact of autophagy on cellular transcriptome. In Arabidopsis, transcriptomic analysis of *atg5* mutant under low nitrate condition revealed that the pathways for glutathione, methionine, raffinose, galacturonate, and anthocyanin are disrupted [32]. However, our present analysis results show that downregulated genes in *ATG5*-silenced pollen were enriched in the pathways related to translation, proton transport, ribosome assembly, whereas upregulated genes in *ATG5*-silenced pollen were enriched in RNA splicing, response to monosaccharide, protein complex biogenesis (Figure S4). There are two potential reasons for the differential downstream pathways of *ATG5* in tobacco and Arabidopsis. The first is the different tissues that were used for transcriptome analysis. Rosettes from Arabidopsis *atg5* mutants were collected under low nitrate stress conditions, while pollen grains were obtained from *ATG5*-silenced plants under normal growth conditions for transcriptome analysis. The second possibility is that the pathways downstream of *ATG5* were indeed not conserved in different plants. Our recent research revealed that autophagy-mediated cytoplasmic clearance is required for tobacco pollen germination, whereas Arabidopsis *atg5* mutant displayed almost normal pollen germination as WT plants [11]. Therefore, whether the role of autophagy and its regulatory pathways are conserved in different plants is worth investigating in the future studies through more detailed analysis.

## 4. Materials and Methods

### 4.1. Plant Materials

*N. tabacum* L. cv. Petite Havana SR1, *ATG2*-silenced and *ATG5*-silenced plants were grown under 12 h of daylight at 25 ± 2 °C in a glasshouse.

### 4.2. RNA Isolation and RNA-Seq

Total RNA was extracted from pollen grains using MiniBEST Plant RNA Extraction Kit (TaKaRa, Kusatsu, Japan). Subsequently, mRNA was purified from the total RNA using Dynabeads^®^ mRNA Purification Kit. Sequencing libraries were constructed using MGIEasy RNA Library Prep Kit according to the reference guide and were subsequently sequenced on the MGIseq2000 platform.

### 4.3. RNA-Seq Data Analysis

The reads containing adaptors and low-quality reads were removed by SOAPnuke [33]. Subsequently, the clean reads were mapped to the tobacco genome and reference transcript sequences (Ntab-TN90 reference Annotation Release 100) using the HISAT2 [34] and Bowtie 2 [35], respectively. The expression level of each gene was quantified as fragments per kilobase of transcript per million mapped reads (FPKM) using RSEM [36]. Differentially expressed genes between WT and *ATG*-silenced groups were identified using DESeq2 [37].

### 4.4. GO Analysis

GO analysis was performed using the public database (https://geneontology.org/, accessed on 30 August 2023). The enrichment *p*-value for each GO term was calculated using a hypergeometric test.

### 4.5. KEGG Analysis

Pathway annotations for tobacco were obtained from the KEGG (Kyoto Encyclopedia of Genes and Genomes) database. The statistical significance of enriched KEGG pathways was determined using a hypergeometric test.

### 4.6. High-Throughput Analysis of Primary Metabolites in Pollen Using GC-TOF/MS

Metabolite extraction for GC-TOF/MS was performed following a previously described method with minor modifications [38]. Pollen from the WT, *ATG2* RNAi, and *ATG5* RNAi plants were collected for high-throughput metabolomics analysis, respectively. Approximately 30 mg of pollen grains was collected and immediately frozen in liquid nitrogen and homogenized using a pestle in a 2 mL tube precooled with liquid nitrogen. Then, each sample was extracted with 0.4 mL extraction liquid (V_methano_l:V_H2O_ = 3:1), and 20 μL of adonitol (1 mg/mL stock in dH_2_O) as internal standard to the sample. Sample extraction, derivatization, and injection were performed as described previously [38]. GC-TOF/MS analysis was performed using an Agilent 7890 gas chromatograph system coupled with a Pegasus HT time-of-flight mass spectrometer. The system utilized a DB-5MS capillary column coated with 5% diphenyl cross-linked with 95% dimethylpolysiloxane (30 m × 250 μm inner diameter, 0.25 μm film thickness; J&W Scientific, Folsom, CA, USA). Metabolites were identified in comparison to a commercial EI-MS library with Chroma TOF 4.3X software of LECO Corporation and LECO-Fiehn Rtx5 database. The RI (retention time index) method was used in the peak identification, and the RI tolerance was 5000. Metabolic features detected in < 50% of QC samples were removed [39]. The resulted three-dimensional dataset, comprising the peak number, sample name, and normalized peak area, was fed to SIMCA14.1 software package for orthogonal projections to latent structures-discriminate analysis (OPLS-DA). To identify differentially expressed metabolites between the WT and *ATG*-silenced pollen, variables with a variable importance in the projection (VIP) value greater than 1 in combination with Student’s *t*-test (*p* < 0.05) were employed.

## 5. Conclusions

In summary, our study employed multi-omics analyses to unveil the pivotal role of autophagy in cellular metabolism during pollen germination. Disrupting autophagy through the silencing of autophagy-specific genes *ATG2* and *ATG5* led to impaired pollen germination. While both *ATG2* and *ATG5* play similar roles in regulating pollen germination, silencing *ATG5* had a more pronounced impact on the transcriptome compared to *ATG2* silencing. Furthermore, our comprehensive high-throughput analysis, which integrates metabolomics, transcriptomics, and proteomics, shed light on potential downstream pathways of autophagy, particularly in amino acid and carbohydrate metabolism, during pollen germination.

## Data Availability

RNA-seq data of WT, *ATG2*-silenced, and *ATG5*-silenced pollen were uploaded to the NCBI Gene Expression Omnibus (GEO) under accession GSE158922.

## Supplementary Materials

The following supporting information can be downloaded at: https://www.mdpi.com/article/10.3390/ijms241915014/s1.
