# Supplementary material for: Integrated Multi-Omics Analyses Reveal That Autophagy-Mediated Cellular Metabolism Is Required for the Initiation of Pollen Germination
Source: Int J Mol Sci. 2023 Oct 9;24(19):15014. doi: 10.3390/ijms241915014 (PMC10573924; doi:10.3390/ijms241915014)
Supplement: Supplementary file 1 [file ijms-24-15014-s001.zip › Supplementary Material.pdf]

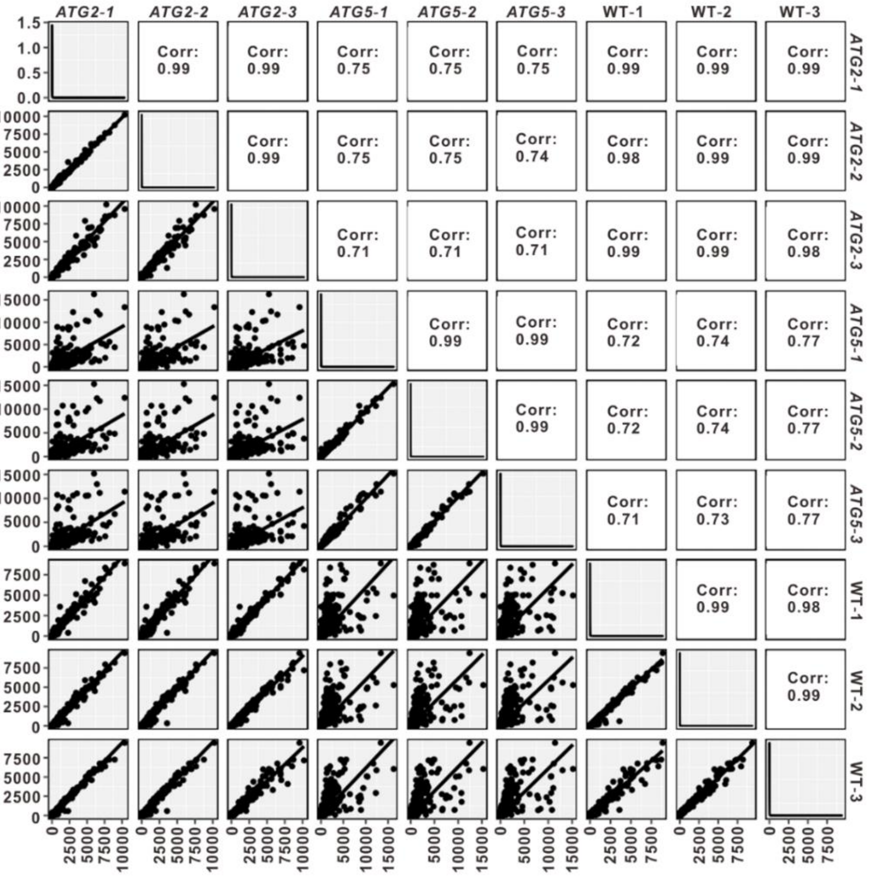

Figure S1. The transcripts from three independent biological replicates are highly correlated

Pearson's correlation coefficients are indicated in the figure.

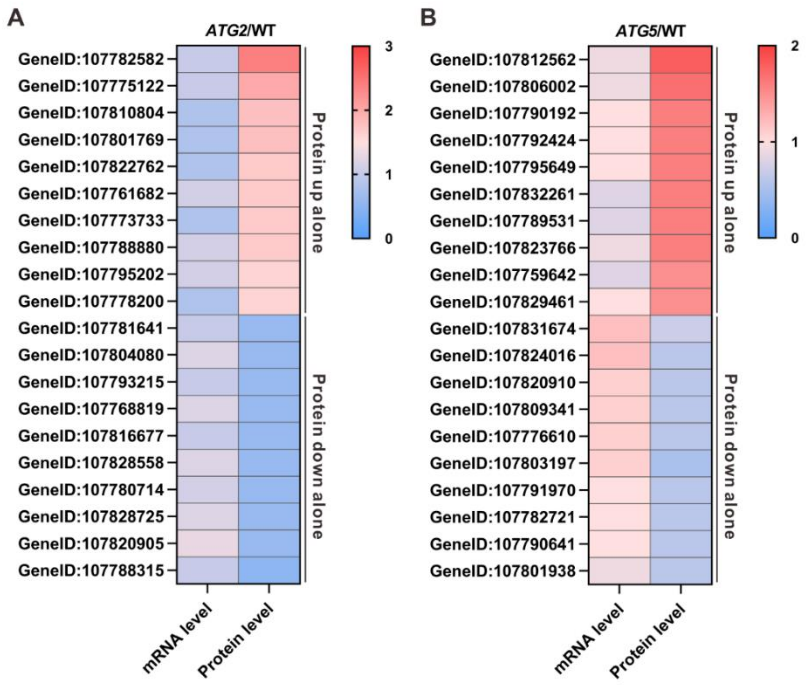

Figure S2. Heat map showing genes that are differentially regulated at mRNA and protein levels

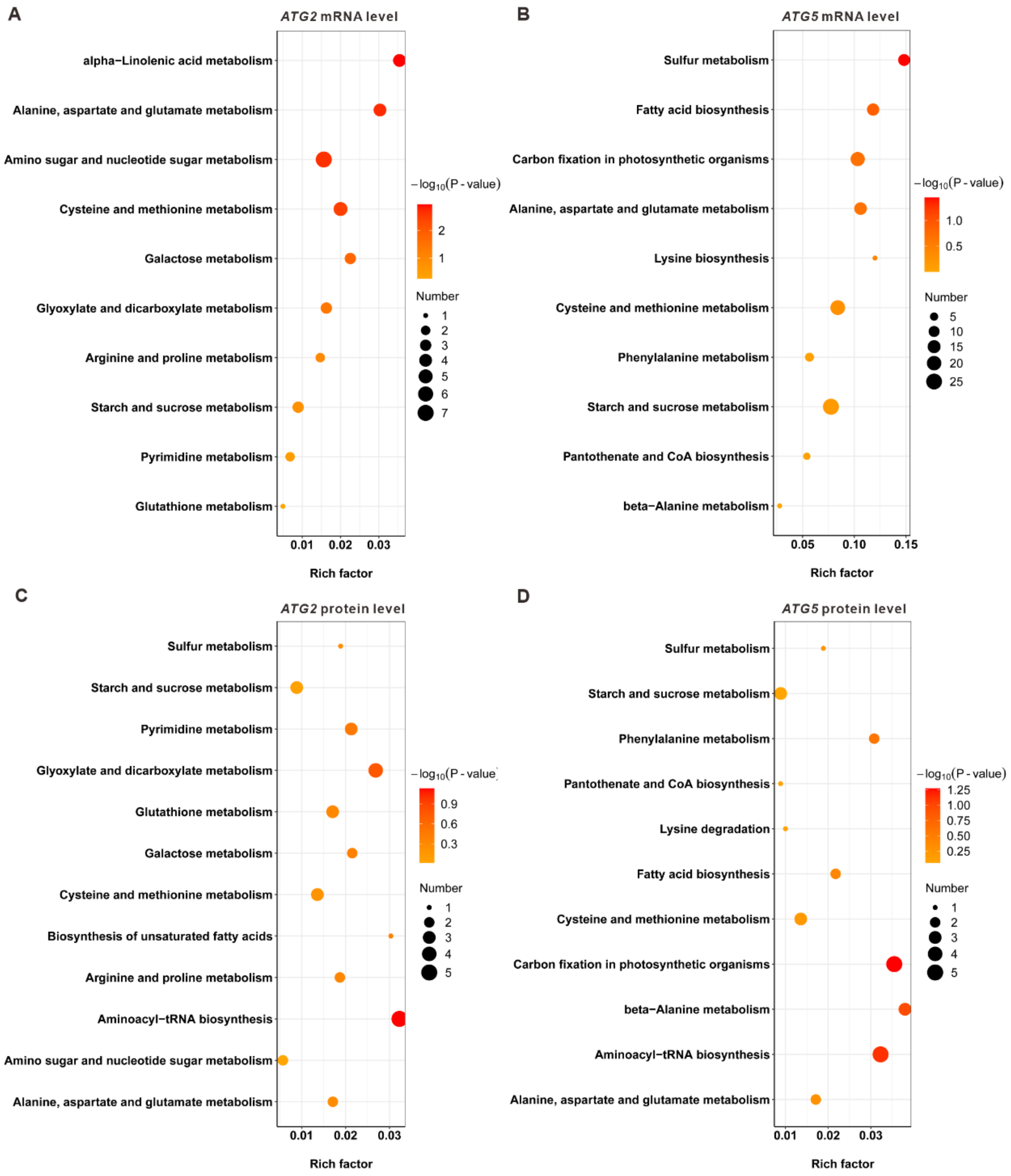

Figure S3. Metabolic pathways identified based on the transcriptomic and proteomic data of *ATG*-silenced pollen.

(A, B). Metabolic pathways affected by the silencing of *ATG2* (A) and *ATG5* (B) during pollen germination were identified based on transcriptomic analysis data. (C, D). Metabolic pathways were identified based on the proteomic analysis data of *ATG2*-silenced (C) and *ATG5*-silenced (D) pollen grains.

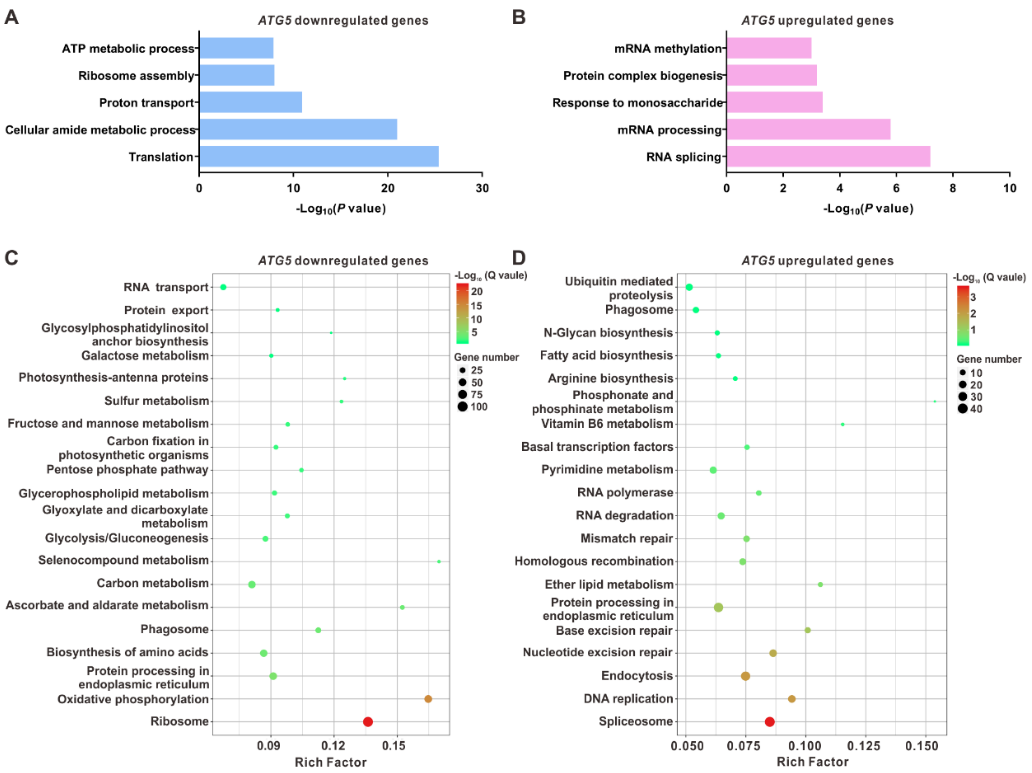

Figure S4. GO and KEGG analysis of differentially expressed genes identified in *ATG5*-silenced pollen.

(A, B). GO enrichment analysis of downregulated (A) and upregulated (B) genes in *ATG5*-silenced pollen. (C, D). KEGG analysis of downregulated (C) and upregulated (D) genes in *ATG5*-silenced pollen.

Table S1. Statistic data of each RNA-seq sample

Sample	Clean Read Pairs	Clean base (bp)	Total mapped reads (ratio)	Unique mapped reads (ratio)	Expressed Genes (FPKM > 0)
WT-1	22,657,803	6,797,340,900	19,881,228 (87.75%)	20,444,056 (90.23%)	41,279 (53.76%)
WT-2	20,933,724	6,280,117,200	18,378,841 (87.80%)	18,965,747 (90.60%)	40,397 (52.61%)
WT-3	25,136,102	7,540,830,600	21,769,750 (86.61%)	22,551,274 (89.72%)	41,973 (54.66%)
ATG2-1	29,438,526	8,831,557,800	25,686,553 (87.25%)	26,418,422 (89.74%)	42,527 (55.38%)
ATG2-2	26,600,116	7,980,034,800	23,234,276 (87.35%)	23,912,599 (89.90%)	42,166 (54.91%)
ATG2-3	23,442,799	7,032,839,700	20,276,792 (86.49%)	20,988,020 (89.53%)	41,453 (52.99%)
ATG5-1	29,653,420	8,896,026,000	25,629,611 (86.43%)	26,726,843 (90.13%)	38,341 (49.93%)
ATG5-2	26,579,411	7,973,823,300	22,765,733 (85.65%)	23,859,858 (89.77%)	39,147 (50.98%)
ATG5-3	27,098,056	8,129,416,800	22,964,257 (84.75%)	24,109,842 (88.97%)	39,119 (50.95%)

Legends for Supplementary Data

Supplementary Data S1. Differentially expressed genes between *ATG2*-silenced and WT pollen or *ATG5*-silenced and WT pollen.

Supplementary Data S2. Gene list used for the comparison of the mRNA and protein levels in *ATG*-silenced pollen.

Supplementary Data S3. Metabolites with different abundances between WT and *ATG2*-silenced pollen or between WT and *ATG5*-silenced pollen.
